# Parent and professional experiences of a clinical trial of prenatal and postnatal stem cell therapy for severe osteogenesis imperfecta

**DOI:** 10.1038/s41431-026-02164-0

**Published:** 2026-06-30

**Authors:** Bikiran Behera, Charlotta Ingvoldstad Malmgren, Eva Åström, Lyn S. Chitty, Belinda Crowe, Anna L. David, Catherine DeVile, Oliver Semler, Magnus Westgren, Cecilia Götherström, Melissa Hill

**Affiliations:** 1 BSc Paediatrics and Child Health, UCL Great Ormond Street Institute of Child Health, London, UK; 2 Center for Research Ethics and Bioethics, Uppsala University, Uppsala, Sweden; 3 Center for Fetal Medicine, Karolinska University Hospital, Stockholm, Sweden; 4 Department of Molecular Medicine and Surgery, Karolinska Institutet, Stockholm, Sweden; 5 Department of Women’s and Children’s Health, Karolinska Institutet, Stockholm, Sweden; 6 Astrid Lindgren Children’s Hospital, Karolinska University Hospital, Stockholm, Sweden; 7 North Thames Genomic Laboratory Hub, Great Ormond Street Hospital for Children NHS Foundation Trust, London, UK; 8 Genetics and Genomic Medicine, UCL Great Ormond Street Institute of Child Health, London, UK; 9 Department of Neurosciences, Great Ormond Street Hospital for Children NHS Foundation Trust, London, UK; 10 Elizabeth Garrett Anderson Institute for Women’s Health, University College London, London, UK; 11 NIHR University College London Hospitals Biomedical Research Centre, London, UK; 12 Department of Pediatrics, University Hospital Cologne, Koln, Nordrhein-Westfalen Germany; 13 Department of Clinical Science, Intervention and Technology, Karolinska Institutet, Stockholm, Sweden; 14 Department of Gynecology and Reproductive Medicine, Karolinska University Hospital, Stockholm, Sweden

**Keywords:** Social sciences, Psychology

## Abstract

Osteogenesis imperfecta (OI) is a rare genetic disorder characterised by bone fragility and frequent fractures. While current treatments aim to preserve bone mass, there is no cure. Boost Brittle Bones Before Birth (BOOSTB4) is an international clinical trial investigating postnatal (*n* = 15) and prenatal plus postnatal (*n* = 3) infusions of fetal mesenchymal stem cells as a potential treatment for severe OI. Each BOOSTB4 participant received four stem cell doses at four-month intervals. Here we explore the experiences of parents and of healthcare professionals involved in BOOSTB4. In this qualitative study, one or both parents of children participating in BOOSTB4 took part after the second dose (T1: 16 families, 22 participants) and at least 6 months after the final dose (T2: 12 families, 16 participants). Professionals delivering BOOSTB4 participated in one interview (*n* = 13). Data were analysed using codebook thematic analysis. Decisions to participate in the trial were influenced by the potential for improved prognosis and limited alternative therapies. Parents felt reassurance because they were taking action and gratitude for access to expertise in OI. Balancing hope, expectations and uncertainty around treatment efficacy was challenging. Parents also expressed disappointment and uncertainty about treatment ending. Practical challenges included travel for treatment with a child with OI or late in pregnancy. Professionals noted the difficulty in managing parental expectations and the regulatory and logistical barriers of an international trial. Findings emphasised the need for clear communication before, during and after the trial, transparent information and trial designs that accommodate the needs of individual families.

## Introduction

OI is a rare condition, clinically and genetically heterogeneous, characterised by osteopenia, growth restriction, bone deformities, fractures and chronic pain. Around 85% of cases result from pathogenic variants in *COL1A1* or *COL1A2*, which encode Type 1 collagen [1]. Clinical presentation varies and was originally classified into four main types: type 1 is the mildest form of OI, type 2 is perinatally lethal, type 3 is a severe form associated with the greatest physical impairment in adulthood, and type 4 is an intermediate group with moderate to severe forms of OI [2]. Severe OI can be detected from early pregnancy using fetal ultrasound when osteopenia, bowing, fractures and shortening of long bones is evident.

The persistent risk of fractures can contribute to significant psychological distress, anxiety, social isolation and lower self-esteem [3, 4]. Children with OI can experience reduced health-related quality of life compared to healthy peers, particularly in physical functioning and emotional well-being [5]. OI burden also extends to parents and families [4, 6, 7]. Ongoing medical care, frequent hospital visits and medical expenses can place considerable strain on family dynamics [4]. Disparities in access to specialised care can exacerbate challenges, particularly in resource-limited settings [8–10].

There are no curative treatments for OI and management focuses on preventing fractures, controlling symptoms and improving function [11, 12]. Bisphosphonates are the primary pharmacological treatment. Bisphosphonates increase bone mineral density by reducing bone reabsorption, but impact on fractures and pain is variable and long-term effects remain uncertain [11, 13]. Other interventions include physiotherapy, occupational therapy and surgical interventions such as intramedullary rodding [14, 15]. Transplantation of fetal mesenchymal stem cells (MSCs) is a potential treatment that could enhance bone regeneration and reduce fracture risk by engraftment of MSCs and production of healthy collagen and bone [16, 17]. Prenatal or early postnatal MSC transplantation may mitigate disease pathogenesis during key phases of skeletal development [17]. Previous qualitative research explored stakeholder views around the hypothetical use of MSC transplantation as a treatment for severe forms of OI [18]. Views were generally positive, and early treatment was considered advantageous for preventing fractures and reducing severity, with psychological benefits for parents. Concerns included procedure-related safety, possible side effects, and challenges for parental decision making when efficacy is uncertain [18]. Some parents may also have concerns around the MSC’s fetal origin [18]. The Boost Brittle Bones Before Birth (BOOSTB4) clinical trial has since evaluated the safety and efficacy of MSCs for severe OI (type 3 or severe type 4) in infants and fetuses [19]. No significant short-term adverse reactions have been identified in infants, pregnant women, or fetuses and indicators such as postnatal fracture rates suggest some positive clinical impacts [20]. In this qualitative study we aimed to explore the experiences of the parents and professionals involved in BOOSTB4 and identify practical barriers and facilitators of trial delivery.

## Methods

### Setting

BOOSTB4 was an exploratory open-label multicentre phase I/II trial that evaluated the safety and efficacy of postnatal (*n* = 15) or prenatal plus postnatal (*n* = 3) transplantation of fetal MSCs for the treatment of severe OI [19]. Trial inclusion and exclusion criteria are summarised in Supplementary Materials. Fetal MSCs were derived from first trimester fetal liver collected from surgical terminations of pregnancy (*n* = 2), from women consented to donate fetal tissues for therapeutic uses. Postnatal participants were aged between 4 months and 16 months at the time of the first MSC dose and received four intravenous doses at four-month intervals. Prenatal plus postnatal participants received the first dose via ultrasound-guided injection into the umbilical vein in the third trimester of pregnancy and three intravenous doses postnatally at four-month intervals. After each dose, in-patient follow-up was 48 (dose 1–2) or 24 (dose 3–4) hours for the postnatal group and 48 (dose 1–3) or 24 (dose 4) hours for the prenatal plus postnatal group. All participants received standard OI treatments including bisphosphonates, physiotherapy and orthopaedic surgery. The Karolinska Institutet sponsored the trial. All doses were administered at the Karolinska University Hospital in Sweden, requiring participants to travel either nationally or internationally from countries including Denmark, Germany, the Netherlands, Norway, Turkey and the United Kingdom to receive each dose. BOOSTB4 commenced in 2020, coinciding with the COVID-19 pandemic.

### Study design

Parents participating in BOOSTB4 were invited to participate in two interviews, approximately 12 months apart, the first (T1) after the second dose and the second (T2) at least 6 months after the final dose. Interviews were also open to parents who declined BOOSTB4, however, no parents declined. Professionals involved in delivering the trial and/or providing clinical care for participating families were invited to participate in a single interview after all trial participants received the second dose. Invited professionals included research scientists; paediatricians and fetal medicine specialists providing clinical care, considering eligibility and discussing BOOSTB4 in Sweden and internationally; and paediatricians, fetal medicine specialists, nurses and midwives administering MSC doses and undertaking monitoring at the trial centre in Sweden.

### Participant recruitment

All parents participating in BOOSTB4 (*n* = 18) were invited to the interview study during their hospital visit for their child’s second dose by a member of the trial team. Parents who provided verbal and written informed consent and were contacted by MH or CIM to schedule the interviews. Professionals were identified with assistance from the trial team and invited to participate via email by MH or CIM.

### Data collection

Interview topic guides explored motivations, decision-making, trial experiences, hopes, expectations, concerns and suggestions for improvements (Supplementary Materials). Interviews were conducted via video conference or telephone by MH (in English, with an interpreter if required) and CIM (in Swedish). Parents could also be sent the questions and provide written responses in any language. Interviews were conducted with one or both parents. Interviews were audio-recorded and transcribed verbatim. Swedish-language interviews and non-English written responses were professionally translated into English. Only English-language content was transcribed in interviews involving an interpreter.

### Data analysis

Transcripts were analysed using a team-based codebook approach to thematic analysis [21, 22]. A codebook approach was chosen to allow a structured approach for individual coding and theme development for the T1, T2 and professional datasets prior to integration. Using a codebook also aligned with the pragmatic choice for two researchers to each undertake coding of different parts of the dataset [23]. Preliminary codebooks were developed deductively based on the topic guides (Supplementary Materials) and our previous research [18], and focused on motivations, decision-making, expectations, psychosocial impacts, trial experiences and practical considerations. Inductive coding was used to incorporate additional codes and concepts. Analysis was facilitated by NVivo14. T1 transcripts were coded by MH, with 20% independently coded by BB. T2 and professional transcripts were coded by BB, with 20% independently coded by MH. Codes were grouped into themes and subthemes that were reviewed and refined (BB, MH). Themes across the three datasets were then compared and integrated to form a single narrative (BB, MH).

## Results

All families (*n* = 18) participating in BOOSTB4 were invited to participate. One or both parents from 16 families were interviewed or shared a written response at T1 between 19/08/2020 and 11/10/2021. T1 interviews lasted 17–58 minutes (median = 39 minutes). One or both parents from 12 families were interviewed at T2 between 09/11/2021 and 10/07/2023, 6 to 11 months after their child’s final MSC dose. T2 interviews lasted 15–60 minutes (median = 28 minutes). Thirteen professionals from fetal medicine, paediatric, research scientist, nursing and midwifery backgrounds were invited to participate, all agreed. Interviews were conducted between 03/08/2022 and 17/11/2022 and lasted 17–53 minutes (median = 37 minutes). Participant characteristics are described in Table 1.

**Table 1:** Participant and data collection characteristics.

Characteristic	Parents T1	Parents T2	Professionals
**Total participants**	22	16	13
**Gender**			
Female	14	9	8
Male	8	7	5
**Country of residence**			
Denmark	1	0	0
Germany	4	2	1
Netherlands	4	4	1
Norway	2	2	0
Sweden	8	6	7
UK	3	2	3
**Profession**	NA	NA	
Fetal Medicine Doctor			4
Fetal Medicine Midwife			1
Paediatrician			5
Research nurse			1
Research scientist			2
**Language for data collection**			
***Interview***			
English	12	11	11
English with translator	2	0	0
Swedish	5	5	2
***Written***			
Danish	1	0	0
German	2	0	0
**Time after MSC dose 2 for data collection**			
0–3 months	13	NA	NA
4–6 months	2		
6–9 months	1		
**Time after MSC dose 4 for data collection**			
6–9 months	NA	8	NA
9–12 months		4	

### Interview findings

**Fig. 1:**
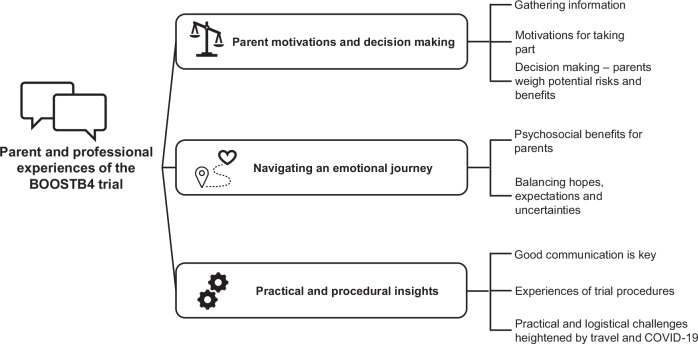
Thematic Map. Themes generated from parent and professional reflections on their experiences of the BOOSTB4 trial.

Themes and sub-themes are summarised in Fig. 1.

#### Parent motivations and decision making

##### Gathering information

Parents first learned about BOOSTB4 from their OI care team, OI support groups or internet searches. Parents reported feeling well informed about the trial before deciding to participate, through discussions with the trial team and their local OI care team and the BOOSTB4 participant information (Table 2: Quote_1). Written information was valued, as parents could return to it later or share with others. Some parents described looking into the results from case studies and animal studies. Several professionals highlighted the value of parents speaking to an “independent informer”, such as a clinician or patient advocate who is not part of the trial team to check understanding and support informed decision making, so that parents “really have understood that this is not something we try to push, but that it’s their own independent decision.” HP08,_fetal medicine doctor (Table 2: Quote_2).

**Table 2:** Illustrative quotes: parent motivations and decision making.

**Gathering information**	
Quote_1	“[name] explained it well to us, told us a lot about what it was about. Then we got the papers to be able to read it through in our own time… The information was good and it was designed well and structured.” P20, mother (T1)
Quote_2	“One way, I think, is of course to inform the parents, I mean to allow someone who is not involved in the Study, or in this sort of treatment activity, to counsel the patient in an independent manner. I think that is quite good. At least it should not be one person alone who does it, because that’s, I think it’s much better than, you work in a group, you do that sort of very, very sensitive passing of information and listening: what is really the wish of this patient or the parents in this situation.” HP12, fetal medicine doctor
**Motivations for taking part**	
Quote_3	“We know that disability, it doesn’t have to mean anything about anyone’s quality of life, so we were kind of level-headed in that, but of course excited if there was something available that could help [child’s name] improve or have better strength.” P01, mother (T1)
Quote_4	“It’s not a fetus. It’s already a baby. It’s already a little person in there moving about so they’ve already got existence. They want to do the best for their child and they feel by taking part that they can do that.” HP03, Fetal medicine doctor
Quote_5	“We can at least make the effort to participate in this trial… maybe it will work, maybe it will not work, at least we did something for him. So, when he’s 20 years old we can explain ourselves to him” P19, mother (T1)
Quote_6	“What I remember is that it can help, and that it is still being researched, and as I understood, in the whole world, it is few who take part.” P04, father (T1)
**Decision making**—**parents weigh potential risks and benefits**	
Quote_7	“So but for some, it has not been an easy decision and for some, I’ve had you know conversations back and forth and had a second meeting, a third meeting.” HP02_Research scientist
Quote_8	“We did have a long list of questions… I mean the participant information lists quite a few potential reactions or side effects – cancer being among them… We were also worried that there was a blind trial, a blind group… But our fears were quite quickly put aside and we weighed the potential benefit of receiving the stem cells were much higher than the potential risks.” P12, father (T1)
Quote_9	“I am very happy not to have to bear the flight and hotel costs myself. The journeys are already exhausting, but doable.” P14, mother (T1)
Quote_10	“The worries are, one, for complications. And then, one, for the child. And then it’s primarily that it will be born or there will be a bleeding in relation to the procedure. Two, for the mother, are there any risks for me as the mother? Can something happen to me? If there will be a birth or a bleeding? Can I be injured by the stem cells or is it dangerous for me? Three, it’s this with the prognosis for the child. How many fractures are there, what do we think it will mean for when the child is born. How well can it be, or how sick can it be? And then, four, how often do we have to come here? And how will we be given more treatments?” HP08, Fetal medicine doctor

##### Motivations for taking part

The most common motivations described by parents for participating in BOOSTB4 were the potential to benefit their child’s well-being (Table 2: Quote_2), early intervention and wanting “to just do everything that we could” (P21, father (T1)) (Table 2: Quote_3). Motivations around potential clinical benefit were sometimes intensified when specific events, such as a series of fractures, had led parents to search for additional interventions.

“During her three first months, she was so little, and still she has gone through seven or six fractures. So, I just felt that all help you can get, I want to take part.” P08, mother (T1)

Feeling responsible for their child’s health was another motivator. Some parents wanted to be able to tell their child they had done something to help (Table 2: Quote_4 and 5). One parent noted that having been repeatedly told about the option to terminate their pregnancy, choosing to continue gave them an “extra responsibility” to give the child a life that is as “good as possible”. Several parents felt treatment options were limited and described feeling “lucky” or “blessed” to be offered an investigational treatment (Table 2: Quote_6). Altruism was also evident, as parents wanted to help other children with OI.

“it’s a win-win situation if we can help people for future for better treatments that’s one thing, and we can give [child’s name] extra chance, he can get better mobility or things like that, whatever the results may be.” P07, father (T1)

##### Decision making—parents weigh potential risks and benefits

Most parents described the decision to join BOOSTB4 as being “easy”, “obvious” or made “quickly”. Professionals noted that whilst many parents decided quickly, for some the decision had not been easy (Table 2: Quote_7). The most concern was the possibility of side effects later in life, with parents ultimately deciding that potential benefits outweighed potential risks (Table 2: Quote_8). Parents and professionals noted that the fetal origin of the MSCs was a major concern for some parents as they were uncomfortable with termination of pregnancy.

“I was concerned about the stem cells actually, not so much that they could harm our kid, but more about where they come from.” P11, mother (T1)

At T2, one parent commented that they had continued to reflect on the source of the cells. Other parents were not concerned about the origin of the MSCs, but felt it was important to be transparent so that parents could “decide themselves.”

Other factors for decision-making included whether the trial had a placebo control group or whether existing treatments such as bisphosphonates would be stopped, both of which would have been a deterrent for participation. Practical considerations around travelling with a child with OI and the costs of travel and accommodation were also described. Having costs paid for or subsidised by BOOSTB4 were valued (Table 2: Quote_9) and influenced some decisions to participate. At T1, parents were asked if they felt pressure to take part from the trial team or local clinicians and all responded that this had not been the case. Some parents noted that they did, however, put pressure on themselves from “inside” to take part and give their child this opportunity.

Additional considerations were raised for the prenatal group. The stress and time pressure of decision-making during pregnancy, the invasive procedure to administer the MSCs, risks for the mother and fetus, travelling to Sweden late in pregnancy and altered birth plans to aid collection of trial samples (Table 2: Quote_10).

“Yeah, it definitely added a degree of stress, you know, so close to the end of the pregnancy, I feel like you’re a little bit under pressure so this was just another thing on top of that to think about and also knowing that if we did say yes we’d have to travel and have quite an invasive procedure.” P3, father (T1)

When reflecting about their decision to participate in BOOSTB4 at both T1 and T2, parents expressed confidence in their choice and said that they would make the “same decision again” or highlighted that they “don’t feel any regret” around the decision.

#### Navigating an emotional journey

##### Psychosocial benefits for parents

At T1 and T2, parents described feeling positive about participating in BOOSTB4 as it allowed them to actively support their child’s health and gave reassurance they were doing “everything we can” (Table 3: Quote_1).

“Yes, it feels nice to say that you’ve done something. You do what you can. It’s nice to know that you’ve done something instead of nothing.” P13, father (T2)

**Table 3:** Illustrative quotes: Navigating an emotional journey.

**Psychosocial benefits for parents**	
Quote_1	“Parents actually felt that they had provided the best possible care for their children. They had the standard treatment but there has been something added and they have actually participated in this trial” HP10, paediatrician
Quote_2	“[Bisphosphonates] is like the standard treatment for her since the 1990s and you always want to do more and there is no more and we are just few of us are able to do something more than just bisphosphonates, so I’m happy that we got the chance to do it and it was worth it” P09, mother (T2)
Quote_3	“So, for the parents to have a better chance of the child going to school and doing other things and not sitting at home or just lying on a bed… So of course it brings huge emotional effects, if it works.” HP08, fetal medicine doctor
Quote_4	“Since I didn’t have to do anything else but being there and watching the ultrasound, it was like, for me it was, yeah, the first time in pregnancy where I really could connect to the kid… I mean, from this point on I was like if the treatment doesn’t do anything, it already has done so much.” P11, mother (T1)
Quote_5	“[Other parents] contacted us to get some information about our experience, so we have been in touch with two or three families in England and Netherlands and Germany” P12, father (T2)
**Balancing hopes, expectations and uncertainties**	
Quote_6	“And we know it’s not a cure, so this is just something we have to accept, that this won’t be a cure in any way, but if it prevents one or two fractures this would be great as well because every fracture, we can, like, yeah, not have is good.” P09, mother (T2)
Quote_7	“But you still want to dare to hope that it will work. But one still needs to have an understanding for the fact that nothing is for sure that it will help; it’s research. But it’s good that there is research, because it helps for the future” P20, mother (T1)
Quote_8	“It was actually like 26 months he didn’t break… so we actually thought it works and now I’m not sure.” P18, mother (T2)
Quote_9	“We had those [parents] who actually very much believed in the trial and some of them also I felt became a little bit disappointed when the trial ended, when they have got their last dose, that they really wanted more… it’s from that they know that they do something potentially good for the child and they are dealt with carefully at the intervals… because they felt that they were actually cared for in a better way than others” HP10, paediatrician
Quote_10	“It’s just like, what happens now, will there be more studies, will there be any follow-up, what happens in other countries… Will it be in a few years that you feel that you have to go to the US and pay a million to see if someone has any stem cells somewhere.” P13, father (T2)

Parents were grateful to access a new treatment (Table 3: Quote_2). Some professionals also noted that the potential benefits could positively influence parents’ emotional well-being (Table 3: Quote_3). For two parents, with OI diagnosed prenatally who were repeatedly asked about termination of pregnancy, participating in BOOSTB4 allowed them to feel more assured that their child was valued by others. One parent was grateful for the requisite monitoring following the prenatal dose which allowed her time and space to connect with her child (Table 3: Quote_4). Several parents valued the trial as an opportunity to access specialist OI care not otherwise available to them.

“Doctor [name] visited us every time we were there, and we got information specifically regarding [child’s name]. I don’t feel anyone else examined her so specifically to know how she is.” P08, mother (T1)

One parent commented that taking part had helped local clinicians to understand the seriousness of her child’s OI, as OI must be “really severe when it is about stem cells” (P13, father (T1)). Another benefit highlighted by some parents was that participating in BOOSTB4 allowed them to meet other parents with similar experiences (Table 3: Quote_5).

##### Balancing hopes, expectations and uncertainties

While all parents entered BOOSTB4 hopeful about the potential benefits for their child, including fewer fractures and less pain, expectations were largely realistic (Table 3: Quote_6). Parents acknowledged that the trial would involve the uncertainty of “waiting and seeing”. Parents also understood that BOOSTB4 aimed to test safety and that there were uncertainties surrounding unknown long-term risks and benefits for their child (Table 3: Quote_7).

Observing progress in their child’s development and long periods without fractures reinforced hope for further improvements and some parents allowed themselves to be more optimistic about the future. However, at both T1 and T 2 many parents described being “afraid of thinking too much ahead” (P5 mother (T2)) and being careful not to raise hopes too high as this could lead to disappointment.

“We don’t know if it’s going to work but maybe it will help, but I’m really like neutral in this whole study, because I don’t want to be overexcited OK this will help him, because I don’t want to be disappointed at the end.” P19, mother (T1)

Parents also worried that a serious fracture could “crush your hopes”. At T2, several parents reported significant “shock” and “disappointment” when their children experienced fractures (Table 3: Quote_8).

Participants described managing uncertainties and changing expectations as the trial progressed. At T2, when parents described improvements in their child, including reductions in fracture frequency, they were careful about attributing improvements exclusively to BOOSTB4. Professionals found it difficult to answer with “complete certainty” parent’s questions on the impact of the trial due to the variable presentation of OI and the impact of other concurrent treatments such as bisphosphonates and rodding.

“If we’re doing things concurrently with our standard care plus the stem cells, what is the additional benefit… it can be challenging to know what’s made the difference.” HP01, paediatrician

Another area of uncertainty and concern for parents was the possibility that the MSCs would not remain effective into the future. Some parents expressed disappointment that the trial was ending, particularly due to the uncertainty surrounding further MSC doses, what to do next to help their child and losing access to specialist OI care (Table 3: Quote_9 and 10).

#### Practical and procedural insights

##### Good communication is key

Parents reported a “good experience overall” and were positive about the communication, treatment and care received throughout the trial (Table 4: Quote_1). There were instances of unclear communication and misunderstandings about trial procedures (Table 4: Quote_2). Language barriers sometimes hindered communication. For example, one professional noted that parents fluent in English were more confident in their trial knowledge (Table 4: Quote_3) and others noted that it could be “difficult” to communicate with parents not fluent in English or Swedish. One parent who did not speak Swedish highlighted that she wanted to understand the discussions of the clinical team that occurred in Swedish so that she could know what was going on and feel more involved. Parents also emphasised their desire for ongoing communication at the conclusion of the trial. Parents wanted transparency regarding the impact of BOOSTB4 on the other children taking part and commented that “summary of study results” or “milestone reports” would be welcomed.

“I’m interested in the outcome and I hope they would keep us informed about it and I hope they will tell us something if there is something we have to know, that they will be open about it” – P09, mother (T2)

**Table 4:** Illustrative quotes: practical and procedural insights.

**Good communication is key**	
Quote_1	“I think it’s been good the way it is and has been. We’ve received the information we need and we’ve got to know what they know and the answers they can give” P20, mother (T2)
Quote_2	“I think the first time we went [after the prenatal dose] with [child’s name] they also wanted to take blood samples from my wife, and I don’t think we were prepared for that, so my wife was a little bit surprised that they needed samples from her as well. I don’t know if the communication was missed or something.” P03, father (T2)
Quote_3	“If you were fluent in English living in a western country, they tended to be more involved in the treatment and the decisions made around the treatment and possible effects of it but maybe if you were coming from another country without the EU community and also if you had that background, they participated because they wanted to but they had not really this confident like knowledge of the trial in that way.” HP10, paediatrician
**Experiences of trial procedures**	
Quote_4	“It was really difficult to spend three days in hospital. But he has felt quite well, actually. And it was difficult with the sting, but it was quite smooth, actually.” P13, father (T1)
Quote_5	“I mean, she developed anxiety about doctors and doctors’ environments… last time that she was there she was very anxious and crying a lot even if they just tried to put the needle in… And also the nurses, they were so nice to her that she reached [a point where] she wasn’t anxious all the time.” P11, mother (T2)
Quote_6	“I wish that all of them had their port-a-cath… because it’s so difficult to put a vein catheter in the hand or in the forehead because they are so sensitive and you are really afraid to have any fracture when you do it.” HP04, research nurse
Quote_7	“It’s a lot of papers to fill out or just concurrently with our standard care to take to the visits and send to us, fill out the child’s diary so maybe that could be sometimes stressful for them.” HP02, research scientist
**Practical and logistical challenges: heightened by travel and COVID-19**	
Quote_8	“Well of course what was a problem was the travel and the journeys for the families that they had to go to Stockholm, yeah, so of course this was not as it was planned, yeah, because definitely was a huge additional hurdle for the trial.” HP11, Paediatrician
Quote_9	“Since I was heavily pregnant, the trip was paid for my partner and me, which was very helpful. Alone I would not have made it in the state of pregnancy.” P06, mother (T1)

##### Experiences of trial procedures

Most parents reported that a stay of several days in hospital could be challenging, but the children had few problems during and after the administration of the MSCs (Table 4: Quote_4). Some parents reported that their child felt highly anxious being in a clinical environment or found the procedure physically uncomfortable or painful (Table 4: Quote_5). Monitoring, such as taking blood pressure and pulse, needed “several times per night”, as part of the trial protocol could also upset the child. One parent suggested finding ways to “measure things without having to wake the child” and recommended attaching the subcutaneous port (port-a-cath) for the MSC infusion while the child is “occupied” to minimise distress (P13, father (T2)). Parents who had the prenatal infusion described the procedure and monitoring as “uncomfortable” but felt fine afterwards.

Not having a port-a-cath for the postnatal doses was a challenging issue described by parents and professionals. Having a port-a-cath was preferred by the trial team, as the alternative of a cannula was thought to be more upsetting for the child and a fracture risk (Table 4: Quote_6). Some parents from countries where port-a-caths are not standard requested a port-a-cath for their child during the trial, which sometimes proved difficult as local clinicians were “really against” it.

The trial also required parents to complete diaries and questionnaires. While some parents found these tasks manageable, others felt some of the questions were “complicated” and it was “difficult to remember” all the required information. Some professionals also highlighted that the required paperwork and questionnaires could be an additional burden (Table 4: Quote_7).

##### Practical and logistical challenges heightened by travel and COVID-19

BOOSTB4 was initially intended to be multi-national, with doses administered in several countries, but delays in regulatory approvals made it necessary for all doses to be given in Sweden.

“This was the first time that really a clinical trial of stem cell transplantation had gone to the regulators… we have not been able to open [in countries other than Sweden] which has been incredibly frustrating because we’ve put a huge amount of work into it.” HP03, fetal medicine doctor

Travel was a barrier for accessibility (Table 4: Quote_8). The capacity of the small trial team in Sweden was stretched and there were practical and logistical challenges with resources and staffing. Parents from countries other than Sweden sometimes described local barriers to obtaining the additional blood and other trial samples. Some parents found that their health service would not support a trial taking place in another country and parents had to organise additional samples themselves and sometimes paid for this to be done privately.

Parents and professionals recognised the unique challenges of travelling with a child with OI, including the risk of fractures, or travelling in late pregnancy (Table 4: Quote_9).

“Yeah there is a lot to organise… So the first time was really, really, really a lot of work to travel with a fragile baby and also to put her through the airport, yeah that was very very difficult but still it’s OK, we managed.” P09, mother (T1)

Organising childcare for siblings was another challenge. The COVID-19 pandemic introduced additional complications as parents had to consider travel regulations and hospital restrictions. Families had to test negative for COVID-19 prior to travel and upon arrival at the hospital. At certain times during the pandemic infection control measures meant that families could not leave the hospital room, or only one family member could be present. Some parent’s had to cancel travel at the last minute or had to go home without treatment.

## Discussion

This study explored parental and professional experiences of a trial for an investigational treatment for OI delivered early in life. Emotional benefits for parents included hope for improvement in their child’s health and well-being, reassurance that they were taking action to help their child and gratitude for access to a team with OI expertise. Other studies have identified advantages to paediatric clinical trial participation that included hopefulness [24], opportunities to help others and positive relationships with study teams [25]. While parents clearly understood that BOOSTB4 was testing safety and benefits were uncertain, they were hopeful that the number of fractures and pain would be reduced. Parents were cautious about embracing hope and some worried that events such as fractures could ruin hope. Parent experiences of balancing hope, expectation and uncertainty have been described in other paediatric clinical trials as a therapeutic optimism that can be challenging for clinical and research teams to manage [26]. Parents need support in managing expectations so that they are prepared for set-backs or no clinical benefits.

The main driver to join BOOSTB4 was the potential for clinical benefit, with many parents motivated by the possibility of improving their child’s health in the context of limited treatment alternatives. This aligns with previous studies where families described trial enrolment as the only viable option and a willingness to try anything [27–29]. Parents felt responsible for helping their child and wanted to do something active to intervene. These findings raise issues for informed consent as decisions can be shaped by perceived clinical necessity. Altruism also influenced parental motivations, mirroring research, where altruism motivated participation in paediatric clinical trials despite uncertain benefit [24, 29]. However, other studies highlight parental reluctance to enrol their own children despite recognising the value of clinical trials [30, 31]. Such variability suggests that altruism is moderated by perceptions of risk–benefit balance and the relevance of the trial to the child’s needs [32, 33].

When parents reflected on decision-making, they reported deliberating potential benefits, uncertainty around long-term risks and logistical factors such as travel and costs. Some parents also reported that deciding to take part would have been more difficult, if bisphosphonates were not permitted or if the trial had a randomised placebo-controlled design. Findings are consistent with studies exploring parental decision-making for paediatric drug trials for genetic conditions where potential for benefits, risk of side-effects, logistics and trial burden have been seen as key factors for decision making [24–26, 34]. Most parents were not concerned about the MSCs being fetal-derived following terminations of pregnancy, though some parents expressed reservations and continued to think about the source of the cells over time. Findings align with paediatric organ transplantation from deceased donors, where families must consider the acceptability of receiving donor-derived tissues and concerns about the source of biological material and personal beliefs similarly influence parental decision-making [35]. The spectrum of stakeholder views on the fetal origin of the cells highlights the importance of transparent, unbiased information to support informed decision-making consistent with personal values [18]. Parent considerations for prenatal MSC administration included the ultrasound-guided invasive procedure. Parents considering postnatal administration rarely highlighted the procedure. This may be because the children already receive bisphosphonates administered in a similar way and have many medical appointments due to fractures and surgery.

The required international travel is typical of rare disease research, where international recruitment is needed to achieve adequate sample sizes. Parents reported the practical burden of travel with a child with OI or in pregnancy and language differences that occasionally hindered communication with the trial team. In addition, professionals described difficulties navigating sponsorship and regulatory processes, which prolonged trial setup and meant that the MSCs could not be administered in multiple countries as originally planned. These findings highlight the complexity of international trial governance and the need for more centralised regulatory frameworks [36, 37].

Parents expressed disappointment at the trial ending and were concerned about stopping regular MSC doses. The loss of specialist care was significant for families where local OI expertise was limited. Similar challenges have been noted in other paediatric trials when relationships with study teams end [24]. Parents requested ongoing updates, including outcome summaries and future treatment options. These findings highlight broader unresolved questions around post-trial responsibilities, particularly who should provide follow-up and to what extent [38]. As trial completion does not end families’ journeys with OI, patient-centred strategies for clear and sustained communication remain essential [39].

A key strength of this study was the inclusion of both parent and professional perspectives. In addition, interview participants were from multiple countries and backgrounds, facilitating the inclusion of diverse experiences and viewpoints. This study has several limitations. Participants were from one clinical trial and wider stakeholder viewpoints are not included. Further, the trial was delivered from a single site entailing international travel for many participants. These are common features of rare disease clinical trials and findings may be transferable to other studies in similar contexts. Not all parents participating in BOOSTB4 took part in the interviews and some only participated at T1 making selection bias possible. Parents who chose not to take part in the interviews were not asked about this decision. Social desirability bias is another potential limitation as parents may feel indebted to the trial team and may have not wanted to describe any negative experiences or criticise the trial. Future research should include the viewpoints of parents who decline trial participation.

## Conclusions

Parent experiences of BOOSTB4 were generally positive and findings offer insights into the practical and psychosocial benefits, concerns and consequences of participation. The study demonstrated the importance of providing transparent information and clear communication regarding clinical trial participation before, during and after the trial. Further consideration of regulatory and logistical barriers in international clinical trials in rare disease research is needed. Addressing these issues will improve the quality of care and support provided to families while enhancing the conduct of clinical research involving OI and other genetic conditions and to ensure a high standard of care if implemented into clinical practice.

## Supplementary information

Interview topic guides

BOOSTB4 Trial Eligibility

## Data Availability

The data that support the findings of this study are available from the corresponding author (MH) where participant consent has been given.
