# Supplementary material for: Parent and professional experiences of a clinical trial of prenatal and postnatal stem cell therapy for severe osteogenesis imperfecta
Source: Eur J Hum Genet. 2026 Jun 30;34(8):1176–84. doi: 10.1038/s41431-026-02164-0 (PMC13424585; doi:10.1038/s41431-026-02164-0)
Supplement: Supplementary file 1 — Interview topic guides [file 41431_2026_2164_MOESM1_ESM.docx]

## BOOSTB4 Interview Study – Topic guides

**Topic guide for interview 1 with BOOSTB4 clinical trial participants**

1. Could you please give me a little background about yourself and your family?
2. Can you tell me about how OI was diagnosed for [name]?
3. What were your thoughts/feelings at the time?
4. How are you feeling about the diagnosis now?
5. Have you been getting the practical and emotional support that you need?
6. What treatments has your child had?
7. Can you tell me about being invited to take part in the BOOSTB4 clinical trial?
   1. What were your first thoughts when you were told about the possibility that your child could have stem cell infusions?
   2. Who approached you?
   3. What did they discuss?
   4. Had you heard of stem cell infusions for OI before?
   5. Were you given any written/printed information?
   6. How much time did you have to make a decision? Was this enough time?
8. What was it like making a decision about taking part in the trial?
   1. What considerations did you weigh up?
   2. What were the main factors that led you to say yes?
   3. What were your main concerns?
   4. Did you have any ethical concerns (fetal origin of cells/research in pregnancy)?
   5. Who did you talk to when making this decision? (partner/family / other health professionals)
   6. What options other than the trial did you feel you had?
   7. Did you feel any pressure from anyone to take part? (e.g. the researchers/health professionals / partner / family members)
9. Do you have any suggestions for how that initial discussion and process of deciding could be improved?
   1. Was anything confusing or unclear?
   2. How good/clear was the written information and consent form?
   3. What additional information would you have liked at that time?
   4. Was the timing of the discussion appropriate for you?
   5. Did you feel supported in your decision-making?
10. How have you found the practical aspects of being involved in the trial?
    1. How has your child been during/after the stem cell infusions?
    2. How have you found the multiple tests / questions etc?
    3. Have you found the travel / multiple visits difficult?
11. What are your hopes for how the stem cell infusions will help your child?
12. Can you see any evidence that makes you think that the stem cell infusions are helping your child?
13. Do you think the stem cell infusions and taking part in the BOOSTB4 trail has had any impact on your relationship with your child or how you feel about their OI?
14. Do you think the stem cell infusions and taking part in the BOOSTB4 trail have had any impact on the relationship between your child and other members of your family?
15. Is there anything else that you would like to add or that you think researchers should consider?

**Topic guide for interview 2 with BOOSTB4 clinical trial participants**

1. Can you tell me a little bit about how [name] is getting on now?
2. Do you feel your confidence has grown when caring for [name]?
3. How are you feeling about the diagnosis of OI now?
4. How does having a child with OI impact on your daily life?
5. How many fractures has your child had in the last 12 months?
6. Have you been getting the practical and emotional support that you need?
7. How did everything go with the last couple of stem cell doses?
8. How do you feel now about your decision to take part in the BOOSTB4 trial?
   1. In hindsight, would you make the same decision again?
   2. What do you see as the benefits for you of taking part?
   3. Have your hopes and expectations changed over time?
9. How have you felt about communication throughout the trial?
10. Have you had enough information from the researchers about the BOOSTB4 trial and about your child’s health over the course of the trial?
11. How have you found the practical aspects of being involved in the trial?
    1. How has your child been during/after the stem cell infusions?
    2. How have you found the multiple tests / questions / diary – interviews etc?
    3. Have you found the travel / multiple visits difficult?
12. What are your hopes and expectations for the outcomes of taking part in the trial? Have they changed over time?
13. Do you think the stem cell infusions and taking part in the BOOSTB4 trail has had any impact on your relationship with your child or how you feel about their OI?
14. Do you think the stem cell infusions and taking part in the BOOSTB4 trail have had any impact on the relationship between your child and other members of your family?
15. What are your hopes for your child in the future?
16. What do you see as the main challenges ahead for your child and your family?
17. Is there anything else that you would like to add or that you think researchers should consider?

**Topic guide for interviews with health professionals**

*For all*

1. Can you tell me about your background and role?
2. Can you describe your experience of working with families affected by OI?
3. What are the main pathways that lead to diagnosis for babies and children?
4. What are the key approaches you use for treatment and therapy for newly diagnosed babies and children?
5. Can you tell me a little about how care for OI is organised in [country]?
6. What are your thoughts on the current treatments and therapies that are available for OI?
7. What has been your role in the BOOSTB4 clinical trial?
8. What are your general thoughts on stem cell transplantation for OI?
   1. What are you expectations of what the main benefits will be?
   2. Do you have any concerns?
   3. Do you feel differently about treatment in pregnancy compared to after birth?
   4. Is offering for the more severe forms of OI appropriate, would you consider broadening it?
   5. Have your thoughts about stem cell transplantation for OI changed over time?
9. [Outside of Sweden] Has your team been involved in recruiting or supporting any families for the trial? What was your role in this?
10. Can you give me a brief overview of the process of recruiting families?
11. What have been the main challenges for identifying families that would be eligible for the trial?
12. [Sweden only] Can you give me a brief overview of how the BOOSTB4 trial is set-up in Sweden?
13. [Sweden only] Have you encountered any practical or organizational difficulties in recruitment or in the day to day running of the BOOSTB4 trial?
14. What have been the main challenges for the BOOSTB4 trial overall?

*For health professionals discussing the study initially and consenting participants*

1. Can you please describe the process of describing the study, supporting decisions and consenting participants?
   1. How long, and over how many appointments, do you spend discussing the trial with parents?
   2. What are the main questions parents ask?
   3. How do you manage parent expectations?
2. Can you tell me about the decision-making process parents go through?
   1. What were the main factors that parents weighed up when deciding about the trial?
   2. What were their main concerns?
   3. Do you think parents felt any pressure from anyone to take part? (e.g. the researchers/health professionals / partner / family members)
   4. Do parents generally give enough time and careful thought to the decision?
   5. Do you think parents general make an informed choice to participate?
   6. What are the barriers to them making an informed choice?
   7. What are the differences for parents making decisions during pregnancy / after birth? Are different approaches to support needed?
3. Have you discussed the trial with any parents who declined to participate? What are the main reasons parents declined to participate? / What are some of the reasons parents might decline?
4. Have you been involved in the care of any of the families after they have had the infusions?
   1. Do you have sense of what their experience of taking part in the trial has been?
   2. What have parent’s expectations been? Are they generally realistic? Is there a need to manage expectations?
   3. Have you noticed any emotional impacts on the parents?
5. Have you seen any clinical benefits that you would attribute to the stem cell infusions in the children taking part in the BOOSTB4 trial?
6. Looking to the future, if stem cell transplantation were to move forward into the next trial phase and ultimately routine care, are there any lessons that you could take from the experience of the trial?
   1. Supporting parents in decision making / during treatment / follow-up care
   2. Practical or organisation issues

*For all*

1. Is there anything else that you would like to add or that you think researchers should consider?
