# Supplementary material for: Parent and professional experiences of a clinical trial of prenatal and postnatal stem cell therapy for severe osteogenesis imperfecta
Source: Eur J Hum Genet. 2026 Jun 30;34(8):1176–84. doi: 10.1038/s41431-026-02164-0 (PMC13424585; doi:10.1038/s41431-026-02164-0)
Supplement: Supplementary file 2 — BOOSTB4 Trial Eligibility [file 41431_2026_2164_MOESM2_ESM.docx]

## BOOSTB4 Trial Eligibility

The inclusion and exclusion criteria for participation in the BOOSTB4 trial are presented in the table below - adapted from Sagar et al 2025. For inclusion, all criteria should apply. For exclusion, one is enough to exclude.

All participants will receive bisphosphonate treatment. At least one dose of bisphosphonate must be administered before the first dose of stem cells in the postnatal group. In the prenatal group, the first dose of bisphosphonates will be administered after birth.

| Inclusion criteria | |
| --- | --- |
| **Postnatal group (n=15)** | **Prenatal group (n=3)** |
| Parent/legal guardian has signed the consent form | Woman has signed the consent form |
|  | Only women where termination of the pregnancy is no longer possible or where the women are committed to continue the pregnancy may be included in the trial |
| Clinical diagnosis of OI type 3 or severe 4  *AND*  Molecular diagnosis of OI (glycine substitution in the collagen triple-helix encoding region of either the *COL1A1* or *COL1A2* gene) | Suspicion of OI type 3 or severe 4 in the fetus on ultrasound findings  *AND*  Molecular diagnosis of OI in the fetus (glycine substitution in the collagen triple-helix encoding region of either the *COL1A1* or *COL1A2* gene) |
| Age less than 18 months | Gestation age between 16+0 and 35+6 weeks+days |
| Parent/legal guardian over 18 years of age | Pregnant women over 18 years of age |
| Exclusion criteria | |
| **Postnatal group (n=15)** | **Prenatal group (n=3)§** |
|  | Multiple pregnancy |
| Existence of other known disorder that might interfere with the treatment, such as, but not limited to organ disfunction (eg, liver or renal failure or bronchopulmonary dysplasia), congenital heart defect, hypoxic encephalopathy l–lll, severe neurological problems, immune deficiencies, muscle diseases, severe malformations or syndromes diagnosed by clinical examination | Coexistence of other disorder that might interfere with the treatment, as judged by the Investigator or the patient’s obstetrician |
| Abnormal karyotype or other confirmed genetic syndrome | Abnormal karyotype or other confirmed genetic syndrome |
| Any contraindication for invasive procedures such as a moderate/severe bleeding tendency | Any contraindication for invasive procedures such as a bleeding tendency or contagious infections, such as, but not limited to HIV, syphilis, hepatitis B, hepatitis C or other known infectious diseases that can harm the fetus |
| Known risk factors for clotting, such as, but not limited to previous blood clot, family history of clots, clotting disorder (inherited or acquired), heart failure, inflammatory disorders (eg, lupus, rheumatoid arthritis, inflammatory bowel disease) | Known risk factors for clotting, such as, but not limited to previous blood clot, family history of clots, clotting disorder (inherited or acquired), heart failure, inflammatory disorders (eg, lupus, rheumatoid arthritis, inflammatory bowel disease) |
| Positive Donor Specific Antibody-test | Positive Donor Specific Antibody-test |
| Known allergy/hypersensitivity to Fungizone and/or Gensumycin | Known allergy/hypersensitivity to Fungizone and/or Gensumycin |
| Oncologic disease (previous or current malignancy) | Oncologic disease in woman or fetus (previous or current malignancy) |
| Inability to comply with the trial protocol and follow-up schedule | Inability to comply with the trial protocol and follow-up schedule |
| Inability to understand the information and to provide informed consent | Inability to understand the information and to provide informed consent |
|  | Unwilling to or cannot undergo delivery by Caesarean section |

**Reference**

Sagar RL, Åström E, Chitty LS, Crowe B, David AL, DeVile C, Forsmark A, Franzen V, Hermeren G, Hill M, Johansson M, Lindemans C, Lindgren P, Nijhuis W, Oepkes D, Rehberg M, Sahlin NE, Sakkers R, Semler O, Sundin M, Walther-Jallow L, Verweij EJTJ, Westgren M, Götherström C. An exploratory open-label multicentre phase I/II trial evaluating the safety and efficacy of postnatal or prenatal and postnatal administration of allogeneic expanded fetal mesenchymal stem cells for the treatment of severe osteogenesis imperfecta in infants and fetuses: the BOOSTB4 trial protocol. BMJ Open. 2024 Jun 4;14(6):e079767.
